# Fc receptors are key discriminatory markers of granulocytes subsets in people living with HIV-1

**DOI:** 10.3389/fimmu.2024.1345422

**Published:** 2024-02-07

**Authors:** Soledad Marsile-Medun, Manon Souchard, Daouda Abba Moussa, Élisa Reynaud, Edouard Tuaillon, Mar Naranjo-Gomez, Mireia Pelegrin

**Affiliations:** ^1^ IRMB, Univ Montpellier, INSERM, CNRS, Montpellier, France; ^2^ Laboratoire de Virologie, Centre Hospitalier-Universitaire de Montpellier, Montpellier, France

**Keywords:** HIV-1, low-density granulocytes, normal-density granulocytes, heterogeneity, PLWH

## Abstract

**Introduction:**

Granulocytes are innate immune cells that play a key role in pathogen elimination. Recent studies revealed the diversity of granulocytes in terms of phenotype and function. In particular, a subset of granulocytes identified as low-density granulocytes (LDG) has been described in physiological conditions and with increased frequencies in several pathological contexts. However, the properties of LDG are still controversial as they vary according to the pathophysiological environment. Here we investigated the heterogeneity of granulocyte populations and the potential differences in phenotype and immunomodulatory capacity between LDG and normal density granulocytes (NDG) in people living with HIV-1 (PLWH).

**Methods:**

To this end, we developed an optimized method to purify LDG and NDG from a single blood sample, and performed in-depth, comparative phenotypic characterization of both granulocyte subtypes. We also assessed the impact of purification steps on the expression of cell surface markers on LDG by immunophenotyping them at different stages of isolation.

**Results:**

We identified 9 cell surface markers (CD16, CD32, CD89, CD62L, CD177, CD31, CD10, CXCR4 and CD172α) differentially expressed between LDG and NDG. Noteworthy, markers that distinguish the two subsets include receptors for the Fc part of IgG (CD16, CD32) and IgA (CD89). Importantly, we also highlighted that the purification procedure affects the expression of several cell surface markers (i.e.CD63, CD66b, …) which must be taken into account when characterizing LDG. Our work sheds new light on the properties of LDG in PLWH and provides an extensive characterization of this granulocyte subset in which Fc receptors are key discriminatory markers.

## Introduction

Neutrophils represent the vast majority (> 95%) of human granulocytes. As part of innate immunity, they have been mostly recognized for their potent antimicrobial effector functions. Through several mechanisms (phagocytosis, NETosis, release of microbicidal and cytotoxic granules, ROS production,…), they act as the first line of defense against invading microorganisms (1, 2). In addition, they are more and more recognized for their ability to interact with innate and adaptive immune cells and to shape the immune response (3). These immunomodulatory functions are expressed through the interaction with numerous immune cells in secondary lymphoid organs (3, 4) as well as through the production of cytokines and chemokines involved in the recruitment and activation of immune cells (5). Neutrophils are thus key immune regulators and actively participate in orchestrating the immune response.

Despite their polyvalent activities, neutrophil granulocytes have been seen for a long time as a homogenous group, whereas they are in fact plastic cells with an intrinsic heterogeneity. Studies in the last decade revealed the diversity of neutrophil granulocytes regarding both phenotypes and functions (6). In particular, a subset of granulocytes is described according to their cellular density lower than usual. These Lower-Density Granulocytes, or LDG, have been described in physiological conditions at very low frequencies (7). However, their frequencies are augmented in several pathophysiological contexts (6, 8). This subset of cells segregates with peripheral blood mononuclear cells (PBMC) after density gradient in contrast to Normal-Density Granulocytes (NDG) which are deposited with erythrocytes (5). Worthy of note, the vast majority of NDG are neutrophils and most of the studies describing the antimicrobial functions of neutrophil above described (ROS production, phagocytosis, NET and granules release, …) concern this type of granulocyte subset.

The first description of LDG dates back to 1986 in systemic lupus erythematosus (SLE), an autoimmune disorder (9). Since then, LDG have been identified in several diseases, such as auto-inflammatory disorders, cancers, bacterial and viral infections including HIV-1 (10–14). Interestingly, LDG display different features in these pathological conditions. However, the functional properties of LDG are still controversial and much less studied than those of NDG, in particular their effector functions mentioned above. On one hand, studies of LDG in tumors showed an ability to inhibit the proliferation and activity of T cells and demonstrated that they act as granulocytic myeloid-derived suppressor cells (G-MDSC) (15). Likewise, LDG are associated with disease severity in SARS-CoV-2 infection, which seems to be related to an immunosuppressive activity (16, 17). On the other hand, LDG seem to be proinflammatory in autoimmune diseases (10). Thus, LDG have mostly been associated with disease-specific immunomodulatory properties. In addition, the maturation status of LDG is still debated, as is the existence of different LDG types/subtypes. Some studies describe LDG as a group of immature cells, but on the opposite, other authors suggest that they are a subset of mature granulocytes. This suggests that the maturation state could depend on the pathophysiological context, and that LDG could be a mixed population comprising different subpopulations. Hence, a more comprehensive vision of LDG might take into account heterogeneity within heterogeneity (18, 19). Thus, LDG abilities and characteristics are, at least partly, dependent of the pathophysiological environment, and seems to vary according to it (20). It is therefore essential to describe this subset of granulocytes in different contexts to better understand its role in the installation or attenuation of a pathology. Toward this aim, there is an undeniable need to identify LDG markers, as they are currently mainly defined by their density (21).

In the case of HIV-1 infection and AIDS-related pathological context, it has been described that LDG are associated with the severity of the infection (14, 22) and could have an immunosuppressive role through the release of arginase-1 (14) and through PD-1/PD-L1 interactions with T cells (23). Nevertheless, LDG are poorly studied and characterized in this viral infectious context. As their frequencies and functions could vary depending on clinical aspects such as immunological or virological status of people living with HIV-1 (PLWH), a rigorous way to identify and isolate them is needed in order to study this subset properly. Our work provides a strategy to purify LDG from PLWH’s blood samples. It also provides an extended characterization of their phenotype with the identification of several markers enabling LDG to be distinguished in whole blood and among purified granulocytes.

## Results

### Strategy for purification of low-density granulocytes from PLWH’s blood samples

**Figure 1:**
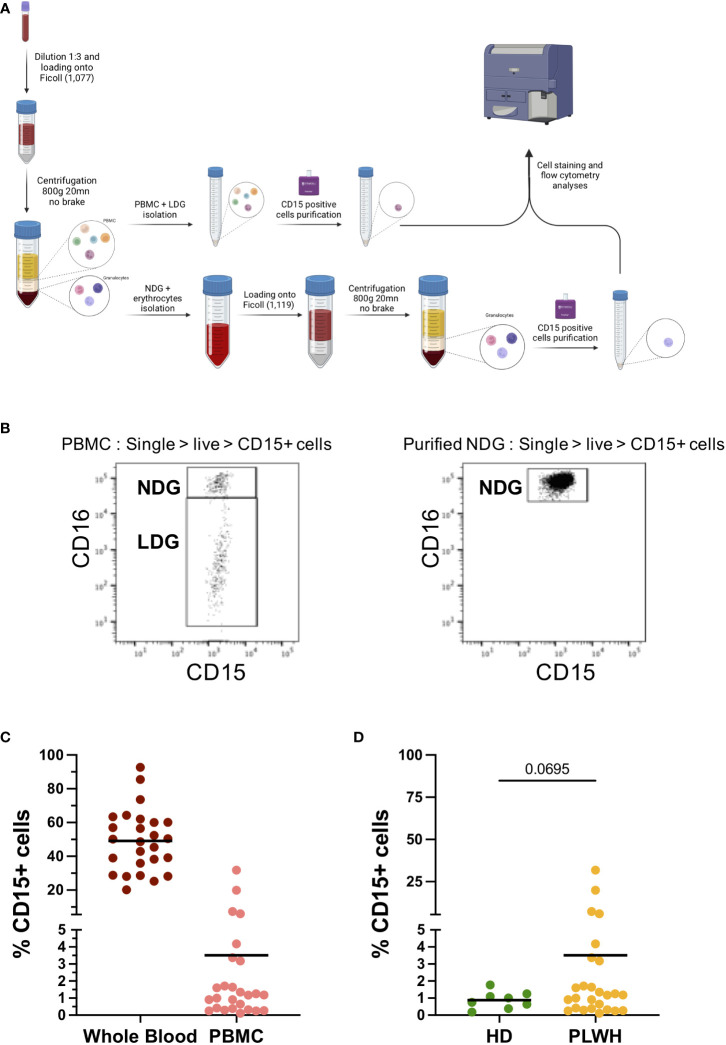
Protocol for isolation of Low-density granulocytes from blood samples and frequencies among PBMC fraction. **(A)** Method for the purification of both NDG and LDG from blood samples. Whole blood is diluted and a first density gradient is done with Histopaque-1077. PBMC phase is collected and CD15^+^ cells are purified by magnetic beads-based technique, while a second gradient is done with higher density cells (granulocytes and erythrocytes), followed by CD15^+^ selection on granulocytic phase. Purified fractions of LDG and NDG were stained and analyzed by flow cytometry. **(B)** Cell repartition according to CD16 expression among PBMC and on purified NDG. CD15^+^CD16^int^ population is absent of NDG purification and thus is defined as LDG. **(C)** Frequency of CD15+ cells among single-live-CD45^+^ cells on whole blood and on PBMC from PLWH samples. Solid line represents mean frequency. **(D)** Compared frequencies of CD15^+^ cells among single-live-CD45^+^ cells on PBMC fractions isolated from PLWH or from healthy donors’ blood samples. Solid line represents mean frequency. Statistical analysis is unpaired t-test with Welch’s correction between LDG and NDG group.

We first established a method to separate and purify both normal-density (NDG) and low-density granulocytes (LDG). Since LDG are currently defined only by their density difference, our method relies on two density gradients. The first one allows the retention of lower density cells among the peripheral blood mononuclear cells (PBMC) using a gradient with a Ficoll of density 1,077. From there, a positive selection of CD15-expressing cells among the PBMC is done to recover LDG. In parallel, granulocytes that have sedimented with erythrocytes are resuspended and loaded on a second Ficoll of higher density (1,119). This second density gradient allows to isolate a second fraction of granulocytes which also undergo a CD15 positive selection to purify NDG (**Figure 1A**). Thus, the succession of two gradients of increasing densities enables a proper separation of low- and high-density cells, i.e LDG and NDG respectively. We rapidly identified CD16 as a discriminating marker between NDG and LDG. Indeed, we found a CD16^int/lo^ cell subset among PBMC (of note, also found in whole blood and purified LDG) whereas this subset was totally absent in purified NDG (**Figure 1B**). For the rest of our study, we used CD16 as a first and robust marker to identify LDG (CD16^int/lo^) in our gating strategy.

Quantification of CD15^+^ cells in whole blood of PLWH showed that granulocytes are, as expected, the main cell type among leucocytes. Among PBMC we found an average of 3,5% of CD15^+^ cells, which correspond to LDG (**Figure 1C**). When compared to the frequency of CD15^+^ cells among PBMC of healthy donors, higher LDG frequencies were observed in one quarter of PLWH blood samples (**Figure 1D**). Worth noting, 4 out of the 25 PLWH showed a frequency of CD15^+^ cells among PBMC ranging from 6 to 30%. Except for gender, as all four samples were from women, we could not identify similarities among the 4 PLWH, neither in term of treatment nor in term of virological and immunological status (**Supplementary Table 1**).

We have therefore established a method to purify LDG and NDG in the blood of PLWH which allows the purification of both granulocyte populations in sufficient quantities to perform an extensive phenotypic characterization.

### Phenotypic characterization of LDG compared to NDG in purified fractions

**Figure 2:**
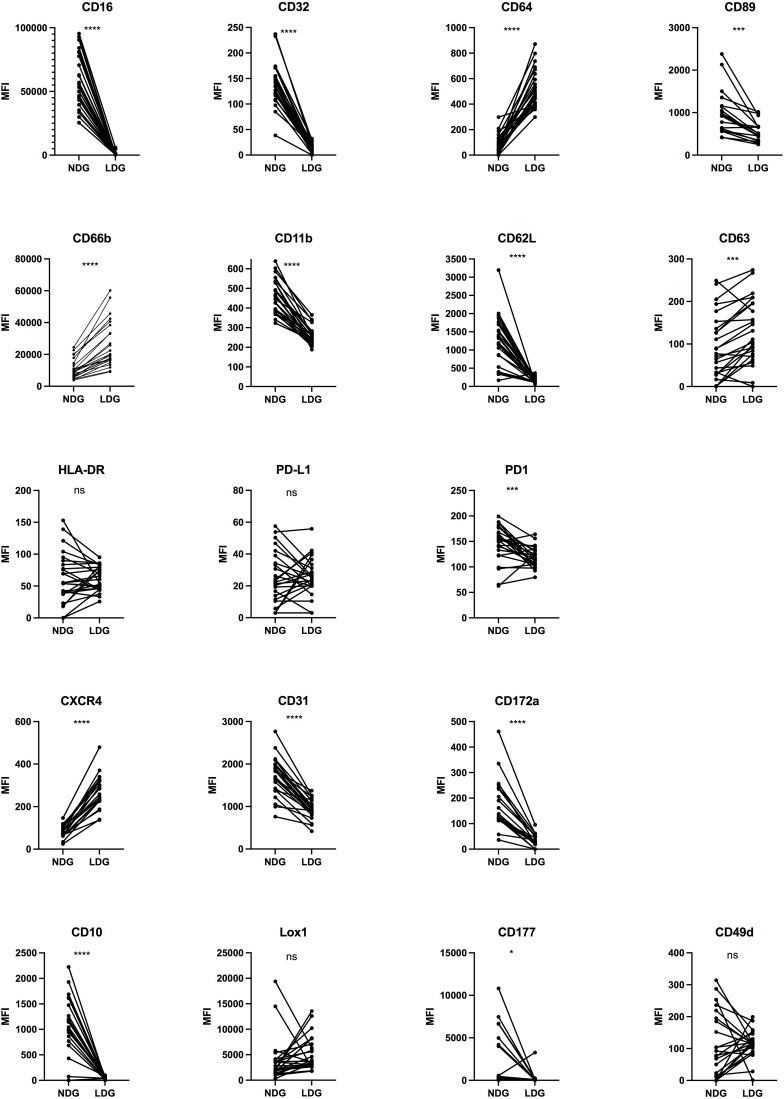
Differential expression of surface markers between purified NDG and LDG isolated from PLWH blood samples. Purified cells were stained for 20 surface markers to assess the phenotype of LDG. Data are the expression of 25 donors except for CXCR4, CD89, CD172α (n=19). Statistical analyses are paired t-test. Significance was assessed as follows: ∗p < 0.05, ∗∗∗p < 0.001 and ∗∗∗∗p < 0.0001. ns: not statistically significant.

We then assessed, on purified fractions of LDG and NDG from PLWH blood samples, the expression of 18 cell surface markers. The panel was chosen to follow the expression of markers of interest in HIV-1 infection (CXCR4), to study neutrophil activation (CD11b, CD66b, etc), immunosuppressive functions (PD-L1/PD-1), maturity (CD10) and Fc receptors (FcRs, CD16, CD32, CD64 and CD89), among others. Some of them were previously described as putative discriminant markers between the two granulocyte subsets in several pathological contexts, including viral infections (see **Supplementary Table 2**). However, to date, there is no consensus either on the kind and combination of markers, nor on the levels of their expression to identify LDG. Among the 18 markers, we highlighted a differential expression between NDG and LDG for 14 of them (**Figure 2**). Particularly, we found that the expression of FcγRs (CD16, CD32 and CD64) and FcαR (CD89) is clearly distinct between both subtypes, with LDG showing a diminished expression of CD16, CD32 and CD89 and, on the contrary, a higher expression of CD64. Regarding the activation markers, LDG showed a lower expression of CD11b and CD177, two cell surface markers that are normally upregulated when cells are activated. However, they displayed also a more activated phenotype with higher expression of CD66b and CD63 (two molecules upregulated at the membrane upon activation) and lower expression of CD62L (a molecule shed during cell activation). Interestingly, adhesion molecules were not augmented, which could have been associated with activation, but were on the contrary, equally (CD49d) or less (CD31, CD172α) expressed by LDG compared to NDG. We also found that LDG do not express CD10, suggesting a more immature state. However, the absence or low expression of CD10 on LDG is still controversial, and some studies found CD10-expressing LDG subtypes (18). Finally, we also identified a higher expression of CXCR4 in LDG.

Overall, our results provide an extended characterization of LDG in PLWH with the identification of several markers differentially expressed between them and NDG. This allows to better identify LDG among leucocytes. In addition, the expression of some of the markers showed a high discrepancy among PLWH (LOX-1, PD-L1, etc), which shows an intrinsic diversity among each subset and highlights another level of diversity for granulocytes.

### Characterization of LDG compared to NDG in whole blood

**Figure 3:**
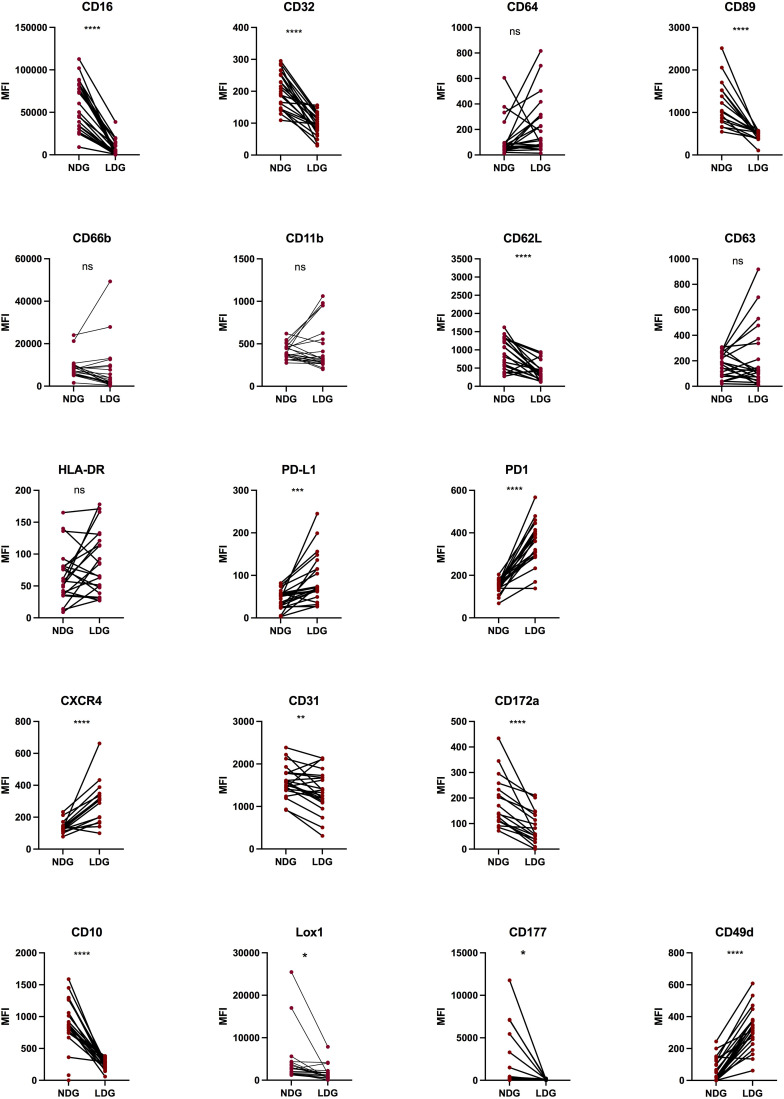
Differential expression of surface markers between NDG and LDG from PLWH blood samples. After erythrocytes lysis, whole blood cells were stained for 18 surface markers to assess the phenotype of LDG previous to purification steps. Data are the expression of at least 20 donors except for CXCR4, CD89, CD172α (n=17). Statistical analyses are paired t-test. Significance was assessed as follows: ∗p < 0.05, ∗∗p < 0.01, ∗∗∗p < 0.001 and ∗∗∗∗p < 0.0001. ns: not statistically significant.

With the identification in purified fractions of a pattern of cell surface markers expression that differs between LDG and NDG, we next assessed if this pattern was already identifiable in whole blood and what impact the purification process had on the expression level for each marker. As LDG show signs of activated cells, the rationale was to determine if this state of activation precedes the purification or is acquired because of it. Thus, we monitored the 18 markers on the blood samples before the first step of purification. We assessed the expression of the markers on single-live-CD45^+^-CD15^+^ cells. We used the expression of CD16 to discriminate between LDG and NDG fractions as this FcγR was strongly differentially expressed between the two purified subsets. Gating in the CD16^int/lo^ population, we found a conserved differential pattern of expression between NDG and LDG for 9 of the 14 markers previously identified (CD62L, CD16, CD32, CD177, CD31, CD10, CXCR4, CD89, CD172α). For other markers, notably cell activation markers (CD66b, CD11b, CD63) and for CD64, the differences between NDG and LDG did not reach statistical significance, showing that changes of their expression might occur during the steps of purification (**Figure 3**). Surprisingly, for PD-1 the expression was different between the two granulocyte subsets, but in a reversed way compared to what was observed on purified cells (increased expression on whole blood LDG instead of decreased expression on purified LDG). Finally, for PD-L1 and CD49d a significant increase in the level of expression was reached between the two subsets which was not the case on purified cells (**Figure 3**). This stronger expression of PD-L1 by LDG is in agreement with previous findings of Bowers and colleagues (23).

These results suggest that the method used to purify the granulocytes is appropriate as the majority of markers identified in purified cells to discriminate LDG from others granulocytes were maintained. However, we observed changes in the expression pattern of some cell surface markers. It appears that the expression of some of them might have been augmented during the purification, such as CD66b and CD63, whereas PD-L1 expression seems to have been lost in the process. Overall, our observations show that LDG can be identified in blood by phenotypic features, and highlight the robustness of FcRs, and particularly of CD16, as identifying markers.

### Evolution of the phenotype of LDG with the purification process

**Figure 4:**
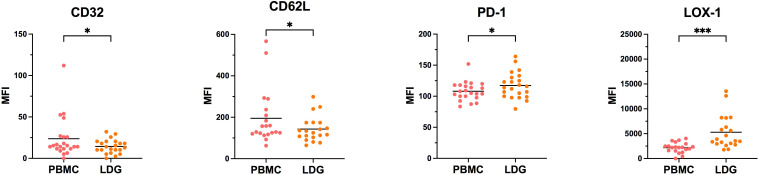
Evolution of LDG phenotype along with the purification process. Cells were stained at the different steps of the purification process to assess the effect of CD15-positive magnetic bead selection on the phenotype of LDG. Data are the expression of at least 20 donors. Statistical analyses are paired t-test. Significance was assessed as follows: ∗p < 0.05 and ∗∗∗p < 0.001.

Considering the differences observed between purified and non-purified LDG from whole blood, we next looked at the evolution of each marker at the two steps of the purification process, i.e PBMC and purified LDG (CD15 positive selection), with a gating in CD16^int/lo^ cells as described above. We observed that for the vast majority of markers, the level of expression was not significantly modified along the second purification process (**Supplementary Figure 1**) with few exceptions (CD32, CD62L, PD-1 and LOX-1). Notably, LOX-1 expression was augmented by the CD15 positive selection step (**Figure 4**). Overall, we showed that the CD15-positive purification step has no major impact on the expression of surface markers of LDG, except for LOX-1 suggesting a susceptibility for this marker to any purification process. On the contrary, gradient-based purification seems to affect the level of expression of some markers, notably activation markers such as CD66b and CD63. Indeed, these markers were upregulated in LDG after the whole purification process (**Figure 2**), with no or few effects of the second purification step (positive selection of CD15) (**Supplementary Figure 1**). Taking into account that whole blood LDG do not show higher expression of these markers than NDG (**Figure 3**), these findings highlight the impact of density-based purification method on LDG phenotype. Overall, these observations could help to interpret phenotypic characterization of LDG depending on the method chosen to obtain these cells.

### Induction of LDG-like phenotype by TLR8 stimulation of healthy donor’s cells

**Figure 5:**
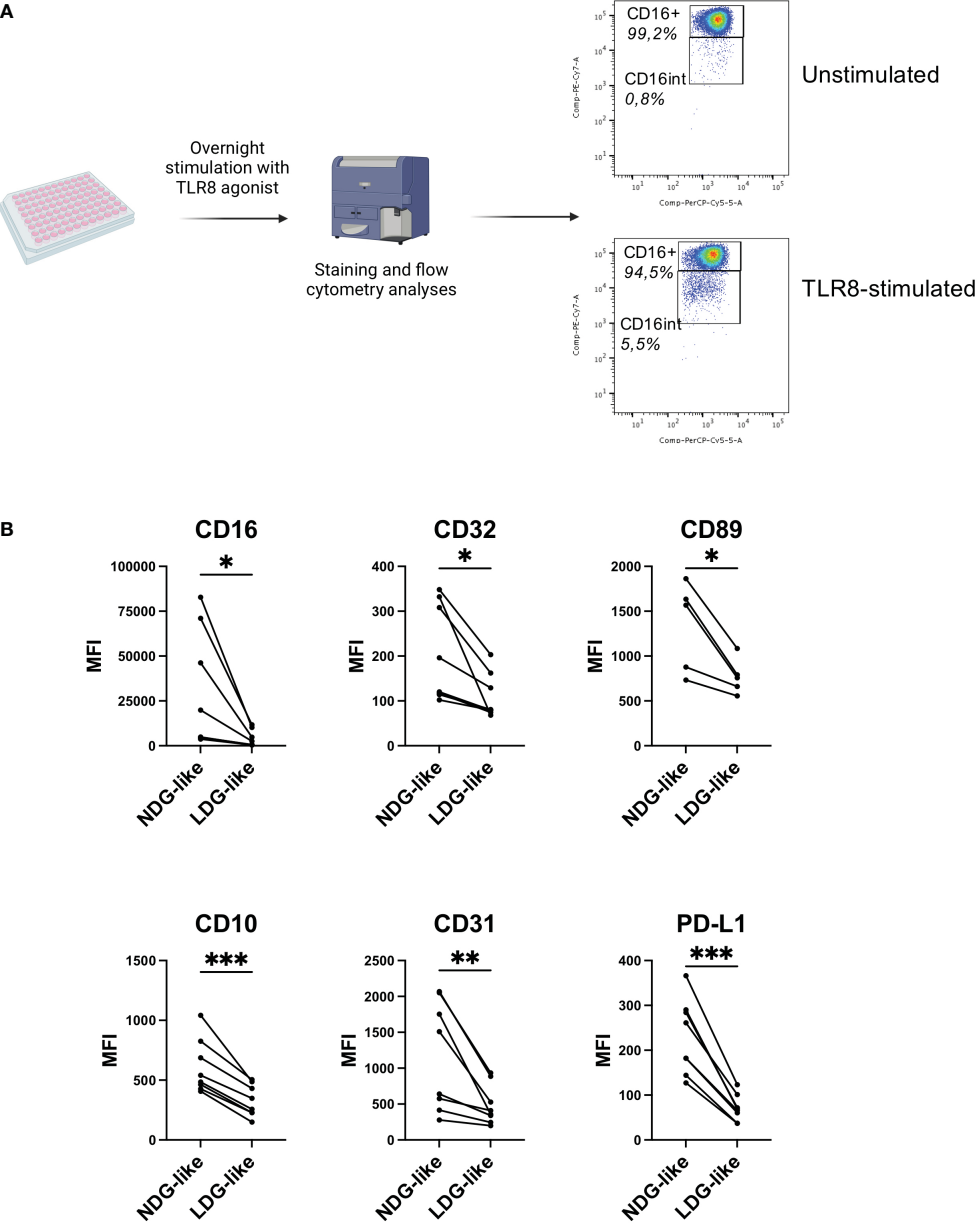
TLR8 stimulation of healthy donors’ granulocytes induces the development of an LDG-like population. **(A)** Purified granulocytes from healthy donors’ blood samples were stimulated overnight with a TLR8 agonist (R848, 0,5 μg/mL) and the phenotype analyzed by flow cytometry. Stimulation induces a population with intermediate expression of CD16. One representative donor is shown. **(B)** Differential expression between LDG-like and NDG-like stimulation-induced granulocytes. Data are from 8 healthy donors. Statistical analyses are paired t-test. Significance was assessed as follows: ∗p < 0.05, ∗∗p < 0.01 and ∗∗∗p < 0.001.

Although the existence of heterogeneity among granulocytes is now recognized, the mechanisms that generate this diversity and its biological relevance remain under debate. One hypothesis for the existence of LDG is that they arise from NDG activated cells (6). It is also described that activated neutrophils (that represents the vast majority of granulocytes) have a decreased cell surface expression of CD16 (24), and we showed that TLR8 stimulation of NDG with the R848 agonist induces the shedding of this FcγR (**Figure 5A**). To test the hypothesis of the induction of an LDG-like phenotype after activation of purified neutrophils, we performed an overnight stimulation of purified NDG from healthy donors with the TLR8 agonist R848 (0,5 μg/mL), mimicking a viral signal of danger. We first confirmed that activation of neutrophils induces a shedding of CD16 for a portion of the cells (**Figure 5A**), leading to a distinction between CD15^+^ CD16^+^ and CD15^+^ CD16^int/lo^ neutrophils, that we called NDG-like and LDG-like respectively. We then assessed for each group the expression of the 18 markers of the panel used for the characterization of PLWH’s LDG phenotype (**Supplementary Figure 2**). Five of the 18 markers (CD10, CD16, CD32, CD89, CD31) were differentially expressed between NDG-like and LDG-like in the same way as NDG and LDG of PLWH whole blood, (i.e. a significant decrease in the expression of these markers on LDG-like *versus* NDG-like) (**Figure 5B**). This supports that these markers allow to identify LDG or LDG-like cells. Moreover, we observed a lower expression of PD-L1 by LDG-like cells compared to NDG-like. Interestingly, this marker was also differentially expressed between LDG and NDG of PLWH, when studied on whole blood, but in the opposite way (higher expression by LDG).

Thus, these results show that it is possible to induce an LDG-like phenotype by activating the granulocytes with a viral stimulus, with notably the modulation of two FcγRs (CD16, CD32) and of the FcαR (CD89) as observed in LDG from PLWH. This suggests that in pathological conditions, the LDG phenotype could result in part from activation caused by the infectious agent or the inflammatory environment associated with infection.

## Discussion

**Figure 6:**
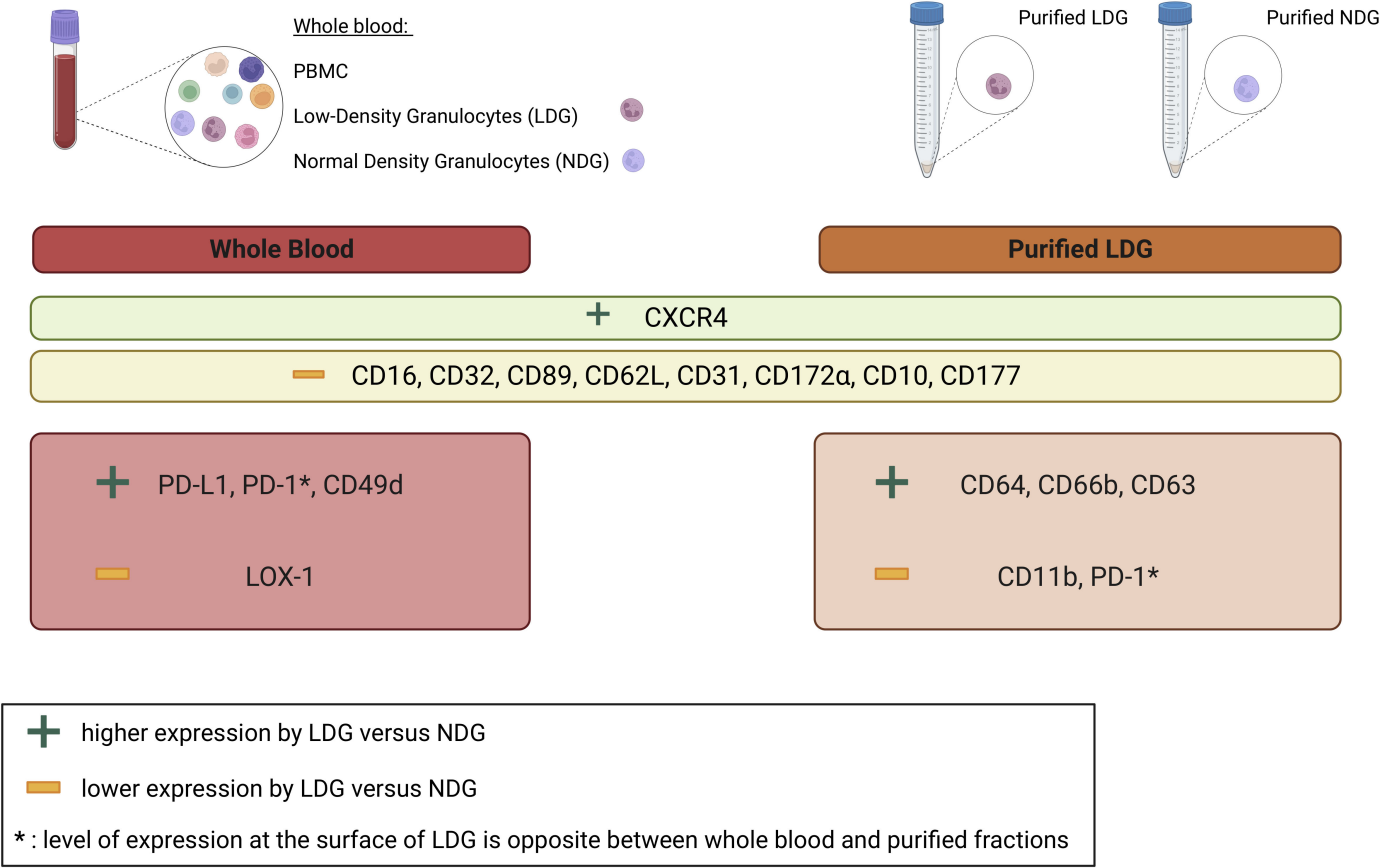
Summary of differentially expressed cell surface markers between LDG and NDG in whole blood and purified granulocytes. Nine cell surface markers are more (CXCR4) or less (CD16, CD32, CD89, CD62L, CD31, CD172α, CD10, CD177) expressed by LDG as compared to NDG in both whole blood and purified fractions. Some markers are also differentially expressed between LDG and NDG in a fraction-specific manner. In particular, higher expression of PD-L1, PD-1, CD49d and a lower expression of LOX-1 by LDG in whole blood and higher expression of CD64, CD66b, CD63 and lower expression of CD11b, PD-1 in purified fractions.

LDG have been described in several pathological contexts in which they exert diverse immunomodulatory roles, either pro-inflammatory or immunosuppressive (6). This diversity highlights the need for a characterization of these cells in numerous diseases in order to better understand the variety of their functions, as well as the need for determining markers to identify LDG without only relying on density difference. Our work provided a method for the purification of LDG in the HIV-1 infection context and we determined an expression profile for cell surface markers that allows the identification of LDG in PLWH, both on purified cells and in whole blood. We also highlighted the impact of the purification process on the level of expression of few cell surface markers. This is of importance as the literature does not reach a consensus on LDG phenotypic characterization, and the purification methods are often scarcely described which is a major factor to explain the disparities between the phenotypes previously described. In this context, it is worth noting the diversity of methods used to obtain and characterize LDG in different pathological contexts (i.e. the diversity of markers studied, the use or not of Heparin and/or EDTA, the purification strategy, the use of fresh or frozen samples,…), has an impact on the observations made. This further highlights the need for standardizing isolation protocols to facilitate granulocytes subset characterization and inter-study comparisons, in line with a very recent study addressing this issue (25). In the particular context of HIV-1 infection, only few studies have focused on the study of granulocyte subpopulations (14, 22, 23). However, such studies involved non-optimized purification process and most of them did not provide a precise phenotype for LDG. Thus, in addition to the methodological advance, our work provides an extensive phenotypic characterization of LDG in PLWH which has not been reported to date (**Figure 6**). Importantly, this is achieved using controlled and optimized approaches.

A major limitation to obtain LDG is that they are a minor population and volume restriction on the blood sample available results in a reduced quantity of cells. However, our purification method allowed us to determine the proportion of LDG among PBMC fraction and to purify enough cells to perform an extensive phenotypic characterization. Importantly, we identified FcγRs and FcαR as discriminating markers between NDG and LDG. In particular, we observed in the latter a decrease in the expression of CD16, CD32 and CD89, but an increase in the expression of the high-affinity receptor CD64. This might affect the ability of granulocytes to respond to natural anti-HIV-1 IgG and IgA or to therapeutic antibodies in the context of antiviral immunotherapies. Worthy of note, a part from CD16 (a FcγRs included in the study of LDG in some, but not all, reported studies), the expression of other FcγRs and FcαR on LDG has not been described thus far, neither in the context of infectious diseases nor in other pathological contexts such as cancer and auto-immune diseases. The reduced expression of CD16 we observed in LDG of PLWH is consistent with a more activated phenotype of this granulocyte subset (26), as is the low expression of CD62L (27). However, other activation markers (i.e. CD11b and CD177) are less expressed by LDG in comparison to NDG, and the upregulation of CD66b and CD63 (two other activation markers) is acquired during the gradient-based purification step). These opposite results do not therefore allow us to conclude on the state of activation of LDGs. It is also important to mention that it is difficult to compare the expression of these cell surface markers under different pathological conditions, as they are not systematically studied [reviewed in (6, 8)]. With regard to the state of maturation, we found signs of an immature phenotype of LDG based on the decreased expression of CD10 and a higher expression of CXCR4 (18, 28). In agreement with this, in the specific context of retroviral infections, low CD10 expression (29) as well as CXCR4 expression (30) has also been associated with an immature state of neutrophils in SIV-infected macaques. In addition, a more recent study highlighted the immature state of LDG in PLWH (31), with notably the identification of a CD16^lo^ LDG subset that resembled granulocyte precursors displaying lower CD10 expression. Moreover, primarily immature CD10^-^ LDG have also been identified in other pathological context such as GVHD (32) and rheumatoid arthritis (33). Thus, our findings suggest that LDG in PLWH are immature cells. However, to formally conclude that LDGs in PLWH are a subset of young immature granulocytes, further investigations might be required.

Our observations also highlight a higher expression of CXCR4 in LDG as compared to NDG (**Figure 6**). Worthy of note, in addition to be a marker of granulocyte maturation, CXCR4 is also important for HIV-1 infection, as it is one of the co-receptors for the virus entry. It is described that HIV-1 can bind to neutrophils in a CD4-independant manner which permits the dissemination of the virus (34). Thus, enhanced CXCR4 expression by LDG could worsen this phenomenon.

We confirmed 9 markers as differentially expressed between LDG and NDG in whole blood samples preceding all purification process (**Figure 6**). Our work thus established an expression profile of cell surface markers that allow to identify LDG without the need for a Ficoll separation. Particularly, LDG are identifiable by their level of expression of FcRs. Similarly, CD16 have been reported in other pathological contexts as a robust marker to identify LDG from NDG (13, 32, 33, 35, 36). However, it is important to take into consideration the discrepancies in the level of expression of CD16 reported in those studies (high, intermediate or low). Surprisingly, the others FcRs were overlooked for the characterization of LDG, although they have emerged from our data as key discriminating factors. Moreover, some of the markers identified could be of importance for the properties of granulocytes, particularly their immunomodulatory roles. Notably, PD-L1, whose expression was lost during the purification process, is related to immunosuppression and its expression was more elevated on LDG in whole blood samples. This further suggests that they are indeed a subset of cells responsible for T-cell function suppression, as previously described (23).

Up to now, there is not a well-defined phenotype of LDG that allows to identify them in peripheral blood. Basically, LDG are obtained through the density gradient due to their differences with NDG. This highlights all the more the high importance of studying LDG and the capital need to find suitable markers in order to study them without the need of density-based purification method. Moreover, some of the markers we identified (CXCR4, PD-L1, PD-1, CD49d) are more expressed by whole blood LDG as compared to NDG (**Figure 3**). This is important as these markers could help to characterize the LDG subset on other criteria than the low or absence of expression of other cell surface markers. It might also be necessary to reconsider the previous studies describing LDG with caution regarding the method used to obtain and characterize LDG. For instance, an activated phenotype could be due to the method used and not to an intrinsic marker of these cells.

In our study we also assessed if the stimulation of granulocytes obtained from healthy donors could lead to the induction of an LDG-like population. We found that TLR8 stimulation of granulocytes induces a CD16^int^ population (LDG-like) that phenotypically differs from the CD16^hi^ cells (NDG-like). This suggests that LDG might be induced, at least partially, by viral-like cell activation. It will be now important to assess whether the induction of this LDG-like phenotype is specific of viral stimulus, or other stimuli, such as pro-inflammatory cytokines or bacterial-like stimuli (i.e. TLR4 agonist) might also induce it. This might allow to better understand the induction of this granulocyte subset in different experimental conditions mimicking the complexity of the pathological environment associated with HIV-1 infection, including virus-driven inflammation and bacterial translocation due to leakage of the intestinal barrier.

Finally, throughout the characterization of LDG from PLWH we noticed an intrinsic heterogeneity inside this subset. To investigate further this second layer of diversity it might be interesting to perform non-supervised analyses on whole blood LDG in order to handle large and complex data without human intervention and reveal novel and unexpected findings.

Overall, our work provides new insight into PLWH’s LDG features and provides an extended characterization of this subset in which FcRs are key markers of discrimination. Moreover, our study supports the hypothesis that LDG are different depending on the environment and consequently confirms the need for an extensive phenotypic characterization of LDG in different pathological contexts with a rigorous methodology in order to better understand the characteristics and origin of this particular granulocyte subset. The ability to identify LDG from whole blood without the need for density-based purification could allow to study these cells in greater depth by transcriptomic analyses. This could be conducted using cell-sorting LDG isolation based on relevant cell surface markers. Mass cytometry could also be used for a better characterization of LDG. This approach has already been used to reveal heterogeneity among simian neutrophils on SIV-infected non-human primates (an animal model for HIV infection) (37). Such a fine technology that allows the study of numerous markers for one cell-type at the same time without the need for fluorescence compensation might be of great interest to perform a deeper characterization of LDG in HIV-1 context, but also on all pathophysiological situations where the presence of LDG is suspected or previously reported. However, the low number of LDG in blood samples is a major limitation for transcriptomic and mass cytometry studies, and will require to have access to a more important volume of PLWH blood samples. The method we established allows the isolation of enough cells to perform flow cytometry, and therefore might be sufficient to use mass cytometry technique.

## Materials and methods

### Ethics

All human blood samples were fully anonymized and provided with previous written consent of the donors. Samples were provided under ethical approval of appropriate institutions.

### Human blood samples

The use of blood samples from anonymized PLWH under antiretroviral therapies (ART) was approved by the Centre for Biological Resources (DRI#305-2019-NC and amendment DRI_2022-69-SR). The samples were collected and provided by the Virology laboratory of the Centre Hospitalier Universitaire (CHU) Lapeyronie of Montpellier (France). Median age is 54 years old ([22;76]) for 25 PLWH donors (15M; 10W). All donors were under successful ART with no detectable viral load. 2 to 3mL of whole blood in EDTA were provided for each PLWH donor. Clinical information of PLWH can be found on **Supplementary Table 1**.

Blood samples from anonymized healthy donors (HD) were provided by the French Establishment for blood donations (EFS; Montpellier, France) according to the agreement between this establishment and INSERM (21PLER2018-0069). EFS provided 7 to 8mL of fresh whole blood in EDTA for each healthy donor.

### LDG and NDG purification

Whole blood sample was first diluted to three times its volume with a solution of PBS 1X (Gibco, cat #10010-015) supplemented with EDTA at 0,2 mM (Invitrogen, cat #15575-038) and loaded on Histopaque-1077 (Sigma-Aldrich, cat #10771). Density gradient was performed by 800g centrifugation with slow acceleration and no brake (acceleration 4, brake 0) for 20 minutes at room temperature. PBMC fraction containing LDG was then collected, washed with a solution of PBS1X supplemented with EDTA at 0,2mM and 2% Fetal Bovine Serum (FBS, Eurobio, REF#CVFSVF00-01) and a CD15-positive selection (Stemcell technologies, CD15^+^ positive selection REF#18651) was performed to retrieve LDG cells. In parallel, granulocytes that have settled along with erythrocytes were resuspended and loaded on Histopaque-1119 (Sigma-Aldrich, cat #11191) and centrifuged for 20 minutes at 800g with slow acceleration and no brake (acceleration 4, brake 0). Granulocytes phase was collected, washed with PBS1X-EDTA 0,2 mM-FBS2% and NDG were purified with CD15-positive selection kit.

### Healthy donors’ primary neutrophils cell culture and stimulation

Purified neutrophils were plated at a concentration of 2 million cells/mL in 96 U-bottom wells plate and cultured in RPMI 1640 supplemented with GlutaMAX™ medium (Gibco, cat #61870-010), 10% fetal bovine serum (FBS, Eurobio, REF#CVFSVF00-01) and 1% penicillin/streptomycin (Gibco, cat #15140-122) with 10 μg/mL of G-CSF (R&D, cat #214-CS-025). For activated conditions, cells were stimulated overnight with TLR8 agonist (R848; Invivogen, cat #tlrl-r848) at 0,5 μg/mL.

### Flow cytometry

Immunofluorescent staining was performed at different steps of the purification process, i.e on whole blood, on PBMCs and on purified LDG and NDG.

For whole blood staining, we first performed an erythrocytes lysis with 1mL of 1X lysis solution for 100 μL of blood (Agilent DAKO, ref#S236430-2) for 15 minutes at room temperature. This process was repeated one time, with intermediate washing step with PBS1X. For all fractions a viability staining was made by resuspending the cells with 1mL DAPI (Biolegend, ref#422801) diluted at 3 μM with an incubation of 15 minutes at room temperature. The cells were then washed in PBS1X and stained with fluorochrome-conjugated antibodies for 20 minutes at room temperature. For the detailed list of antibodies and suppliers see **Supplementary Table 2** (38–42). After the washing of fluorescent antibodies, cells were fixed with 100 μL PFA4% for 10mn at room temperature. Finally, cells were washed and resuspended with PBS1X. Data acquisition was performed on a flow cytometer BD LSRFortessa (BD Biosciences). Forward scatter area and forward scatter height as well as side scatter area and height were used to remove doublets from flow cytometry analyses. Data were analyzed using FlowJo software version 10.5.3 (TreeStar).

### Statistics

Statistical analyses were performed using Prism software version 9.5.1 (GraphPad). Simple group comparisons were performed using paired t-test. Multiple group comparisons were performed using Repeated-Measures one-way ANOVA. Significance was assigned as follows: ∗p < 0.05, ∗∗p < 0.01, ∗∗∗p < 0.001, and ∗∗∗∗p < 0.0001.

## Data availability statement

The raw data supporting the conclusions of this article will be made available by the authors, without undue reservation.

## Ethics statement

The studies involving humans were approved by Centre for Biological Resources; CHU Montpellier. The studies were conducted in accordance with the local legislation and institutional requirements. The human samples used in this study were acquired from a by- product of routine care or industry. Written informed consent for participation was not required from the participants or the participants’ legal guardians/next of kin in accordance with the national legislation and institutional requirements.

## Author contributions

SM-M: Conceptualization, Data curation, Formal analysis, Funding acquisition, Investigation, Methodology, Validation, Writing – original draft. MS: Data curation, Investigation, Methodology, Writing – review & editing. DAM: Data curation, Investigation, Methodology, Writing – review & editing. ÉR: Resources, Writing – review & editing. ET: Resources, Writing – review & editing. MN-G: Conceptualization, Data curation, Formal analysis, Funding acquisition, Investigation, Methodology, Supervision, Writing – original draft. MP: Conceptualization, Formal analysis, Funding acquisition, Investigation, Methodology, Project administration, Supervision, Writing – original draft.

## Supplementary material

Supplementary Figure 1 Evolution of LDG phenotype along with the purification process. Cells were stained at the different steps of the purification process to assess the effect of CD15-positive magnetic bead selection on the phenotype of LDG. Data are the expression of at least 20 donors. Statistical analyses are paired t-test. Significance was assessed as follows: *p < 0.05, and ***p < 0.001.

Supplementary Figure 2 Differential expression between LDG-like and NDG-like TLR8 stimulation-induced granulocytes. Data are from 4 to 8 healthy donors. Statistical analyses are paired t-test. Significance was assessed as follows: *p < 0.05, **p < 0.01 and ***p < 0.001.
