# Supplementary figures and images for: Fc receptors are key discriminatory markers of granulocytes subsets in people living with HIV-1
Source: Front Immunol. 2024 Feb 7;15:1345422. doi: 10.3389/fimmu.2024.1345422 (PMC10879334; doi:10.3389/fimmu.2024.1345422)

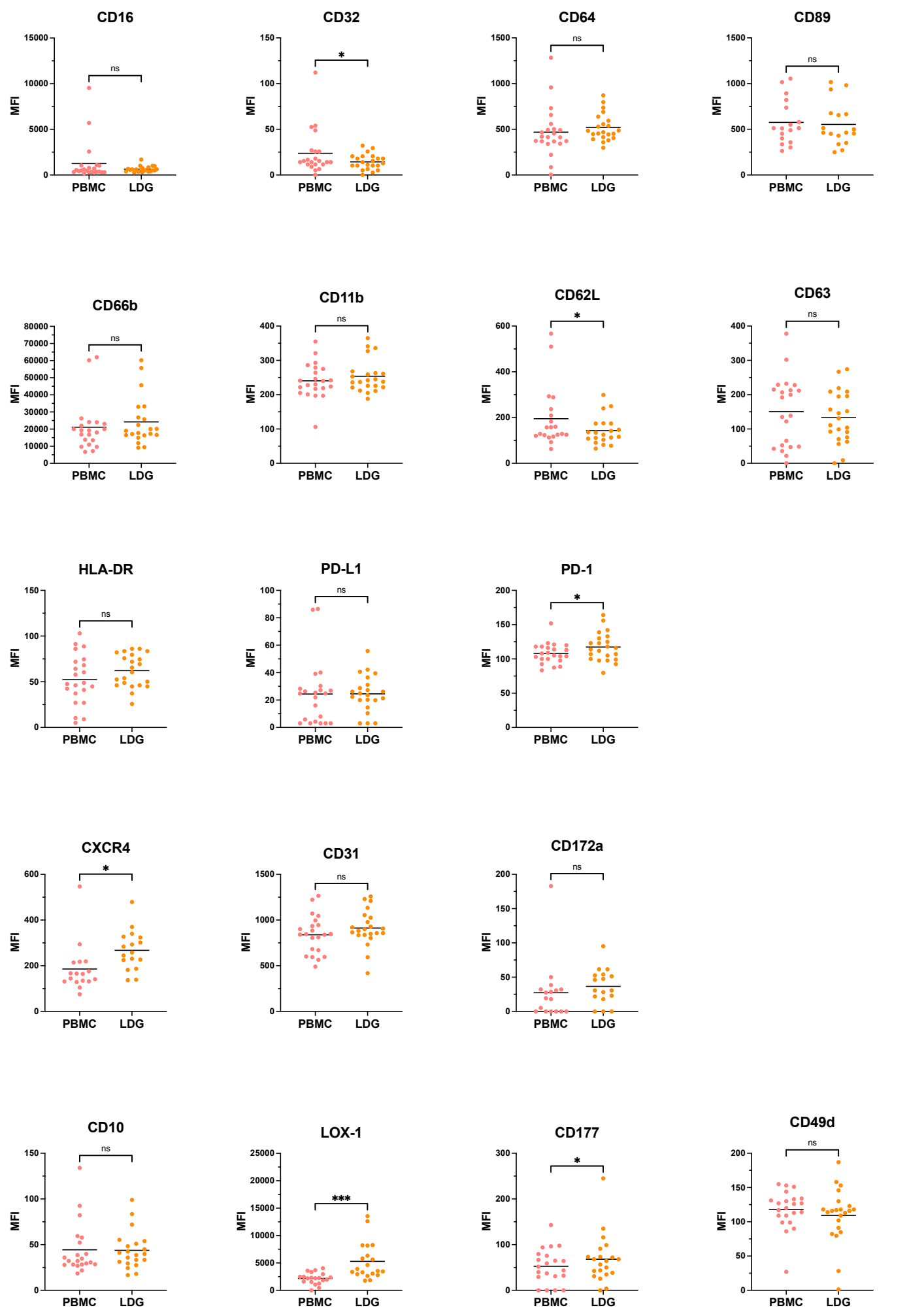

Supplement: Supplementary Figure 1 — Evolution of LDG phenotype along with the purification process. Cells were stained at the different steps of the purification process to assess the effect of CD15-positive magnetic bead selection on the phenotype of LDG. Data are the expression of at least 20 donors. Statistical analyses are paired t-test. Significance was assessed as follows: *p < 0.05, and ***p < 0.001. [file Image_1.pdf]

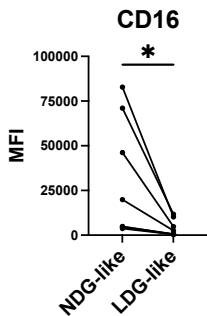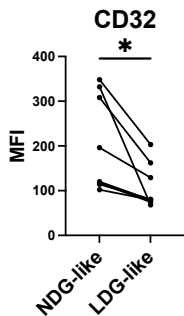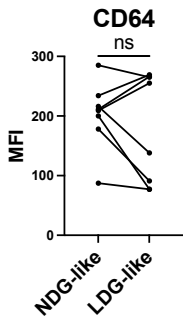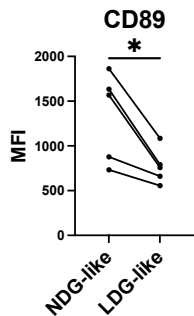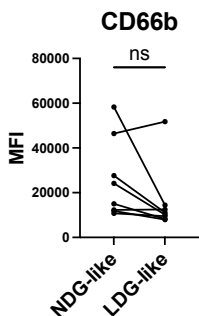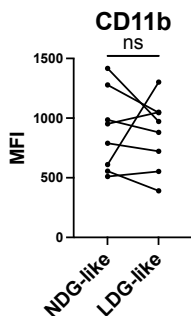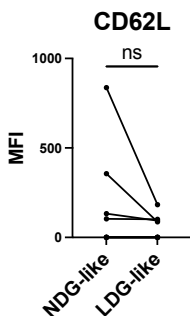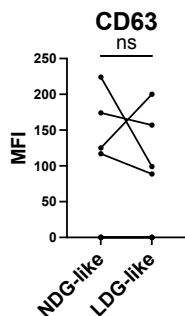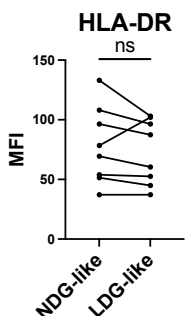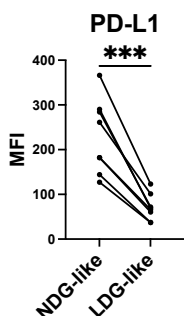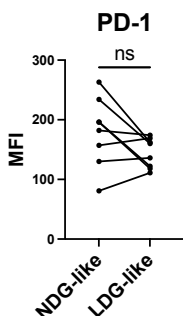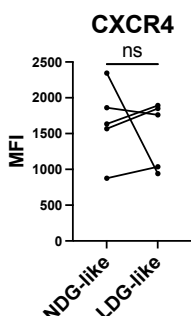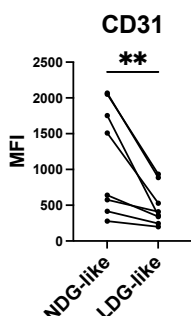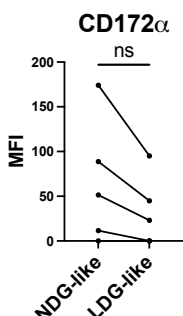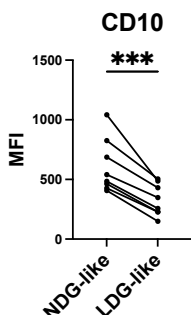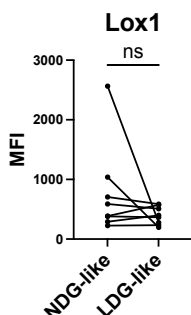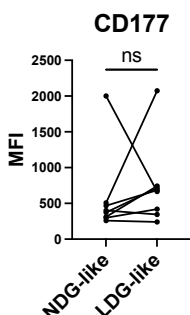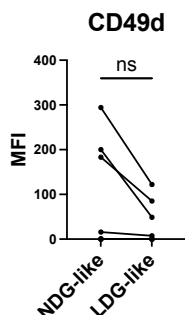

Supplement: Supplementary Figure 2 — Differential expression between LDG-like and NDG-like TLR8 stimulation-induced granulocytes. Data are from 4 to 8 healthy donors. Statistical analyses are paired t-test. Significance was assessed as follows: *p < 0.05, **p < 0.01 and ***p < 0.001. [file Image_2.pdf]
