# Supplementary material for: Fc receptors are key discriminatory markers of granulocytes subsets in people living with HIV-1
Source: Front Immunol. 2024 Feb 7;15:1345422. doi: 10.3389/fimmu.2024.1345422 (PMC10879334; doi:10.3389/fimmu.2024.1345422)
Supplement: Supplementary file 3 [file Table_1.docx]

**Supplementary Table 1: Demographic and clinical data of ART-treated PLWHIV cohort.** DOL: Dolutegravir; ABC: abacavir; 3TC: lamivudine; TAF: tenofovir alefenamide; FTC: emtricitabine; BIC: bictegravir; RAL: raltegravir; DRV: darunavir; RTV: ritonavir; EVG: elvitegravir; Cobi: cobicistat; LPV: lopinavir; TDF: tenofovir; NVP: nevirapine; ETR: etravirine; RPV: rilpivirine; CAB: cabotegravir. Suffix -LA means the antiretroviral comes as injections.
