# Supplementary material for: Fc receptors are key discriminatory markers of granulocytes subsets in people living with HIV-1
Source: Front Immunol. 2024 Feb 7;15:1345422. doi: 10.3389/fimmu.2024.1345422 (PMC10879334; doi:10.3389/fimmu.2024.1345422)
Supplement: Supplementary file 4 [file Table_2.docx]

**Supplementary Table 2:** Surface markers studied by flow cytometry on low-density granulocytes. Clones and fluorochromes for the flow cytometry antibodies are indicated as well as the supplier catalog number. This panel was decided to assess several markers susceptible to help to identify LDG from NDG in various pathological contexts according to the literature.

| Marker | Fluorochrome | Clone | Supplier | Reference |
| --- | --- | --- | --- | --- |
| *CD45* | BV786 | HI30 | Biolegend (#304048) |  |
| *CD15* | PerCPCy5-5 | HI98 | Biolegend (#301922) |  |
| *Siglec-8* | PeCY5 | 7C9 | Biolegend (#347114) |  |
| *CD123* | Alexa Fluor 700 | 6H6 | Biolegend (#306040) |  |
| *CD14* | FITC | MoP9 | StemCell Technologies (#60124FI) |  |
| *CD4* | BV605 | RPA-T4 | Biolegend (#300555) |  |
| *CD19* | BV650 | HIB19 | Biolegend (#302237) |  |
| *CD56* | BV711 | 5.1H11 | Biolegend (#362541) |  |
| *CD3* | PE-CF594 | HIT3a | Biolegend (#300335) |  |
| *Lox-1* | PE | 15C4 | Biolegend (#358604) | (35,38,39) |
| *CD11b* | BV510 | ICRF44 | Biolegend (#301334) | (22) |
| *CD66b* | Alexa Fluor 647 | G10F5 | Biolegend (#305110) | (20,22) |
| *CD62L* | BV605 | DREG-52 | Biolegend (#304834) | (23,40) |
| *HLA-DR* | BV650 | L243 | Biolegend (#307650) | (41) |
| *CD63* | BV711 | H5C6 | Biolegend (#353042) | (38) |
| *CD16* | PC7, BV510 | 3G8 | Biolegend (#302016, #302048) | (16,35,32) |
| *CD64* | PE-CF594 | 10.1 | BD (565389) |  |
| *CD32* | APC-Fire450 | FUN-2 | Biolegend (#303220) |  |
| *PD-1* | PE | 3.1.3 | Miltenyi (#130-177-384) |  |
| *CD177* | APC | MEM-166 | Biolegend (#315808) | (35,36) |
| *CD49d* | BV605 | 9F10 | Biolegend (304324) |  |
| *CD31* | BV711 | WM59) | Biolegend (#303136) | (42) |
| *PD-L1* | PC7 | MIH1 | BD (#558017) | (38) |
| *CD10* | PE-CF594 | HI10a | Biolegend (#312227) | (19,35) |
| *CD89* | PE |  | BD (#555686) |  |
| *CD172α* | APC | 15-414 | eBiosciences (#17-1729-42) |  |
| *CXCR4* | PC7 | 12G5 | Biolegend (#306514) | (23) |
